# Supplementary material for: Characterization of Antimicrobial, Antioxidant, and Leishmanicidal Activities of Schiff Base Derivatives of 4-Aminoantipyrine
Source: Molecules. 2019 Jul 24;24(15):2696. doi: 10.3390/molecules24152696 (PMC6696115; doi:10.3390/molecules24152696)
Supplement: Supplementary file 1 [file molecules-24-02696-s001.pdf]

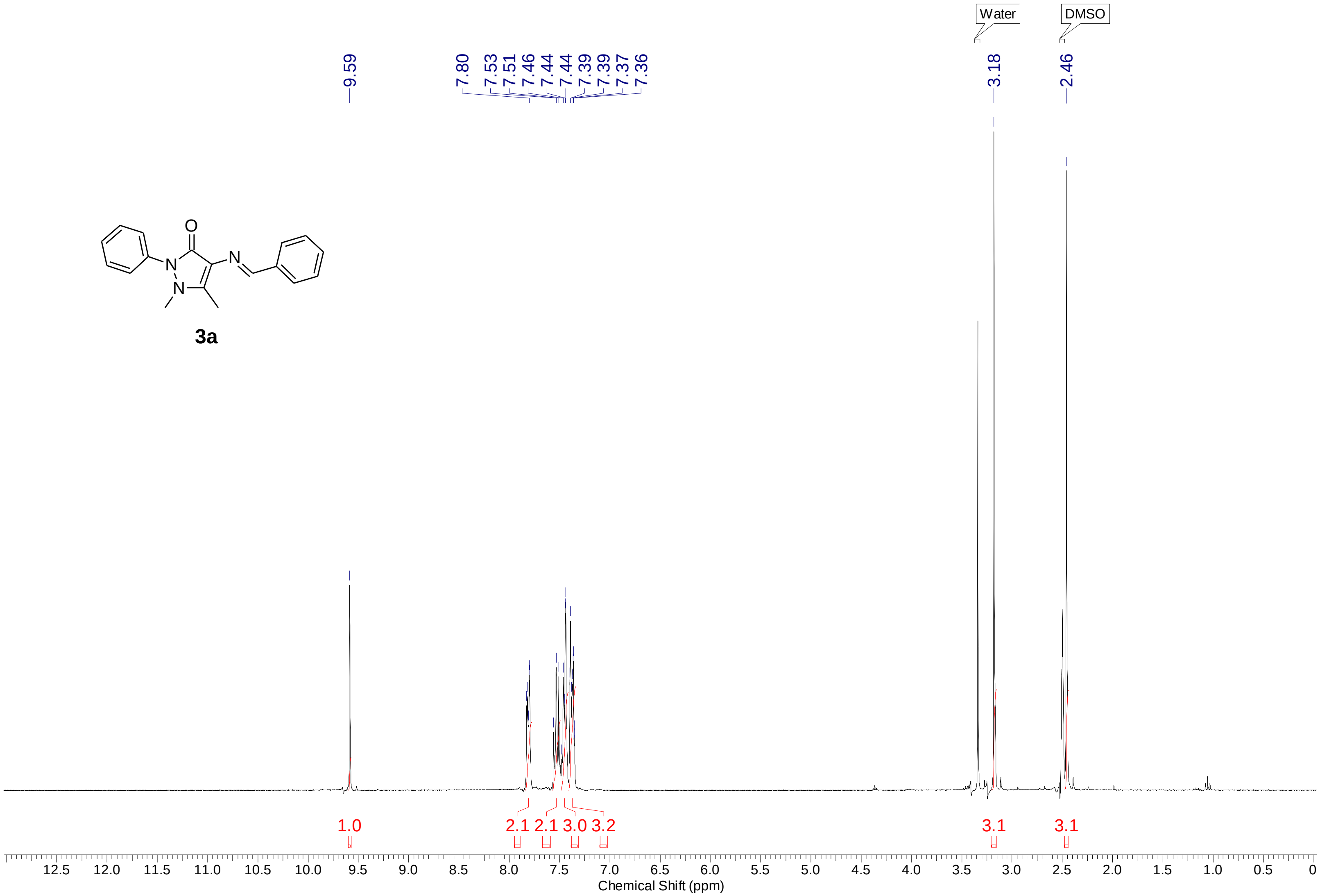

Figure S1. ^{1}H NMR spectrum of compound **3a**.

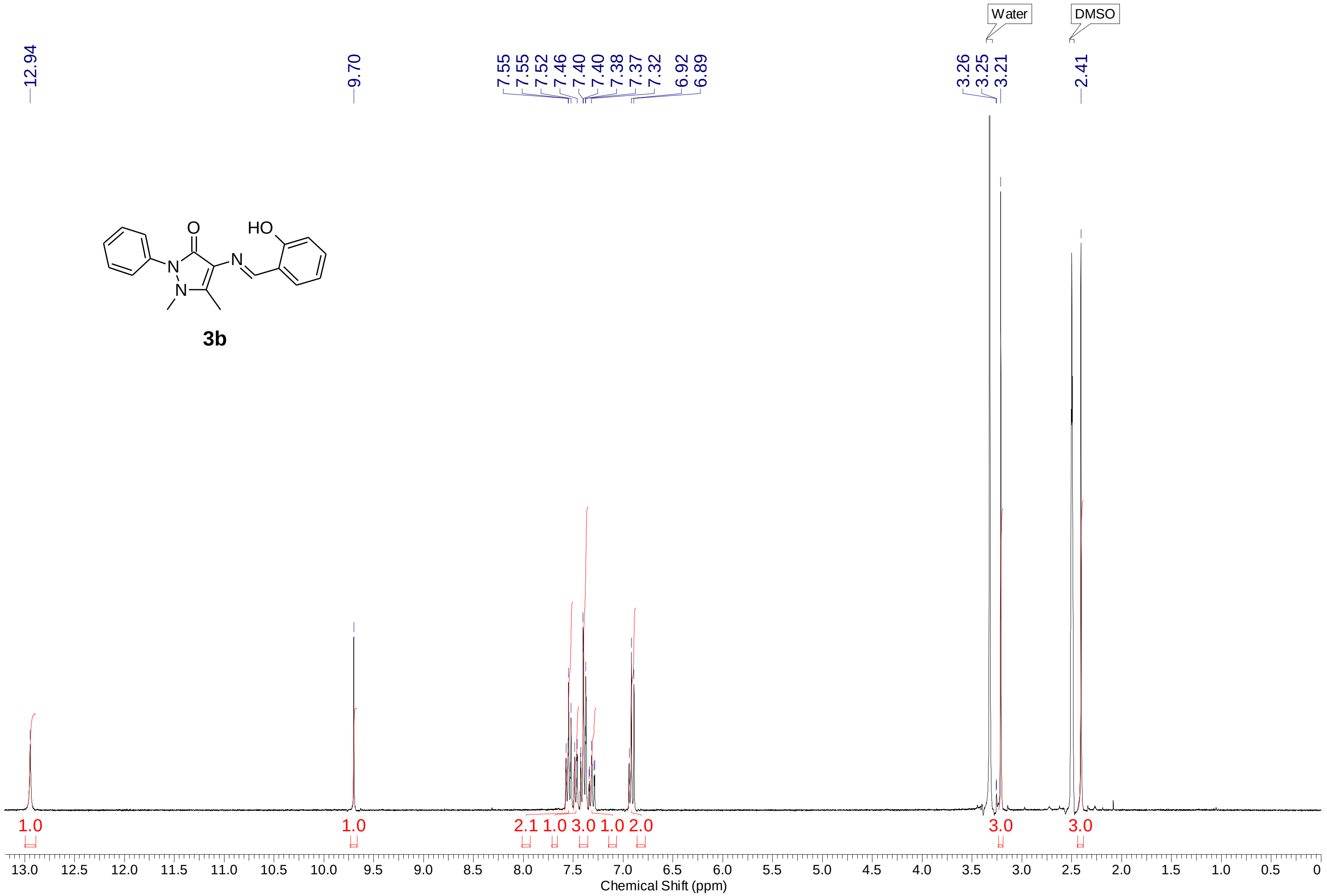

Figure S2. ^{1}H NMR spectrum of compound **3b**.

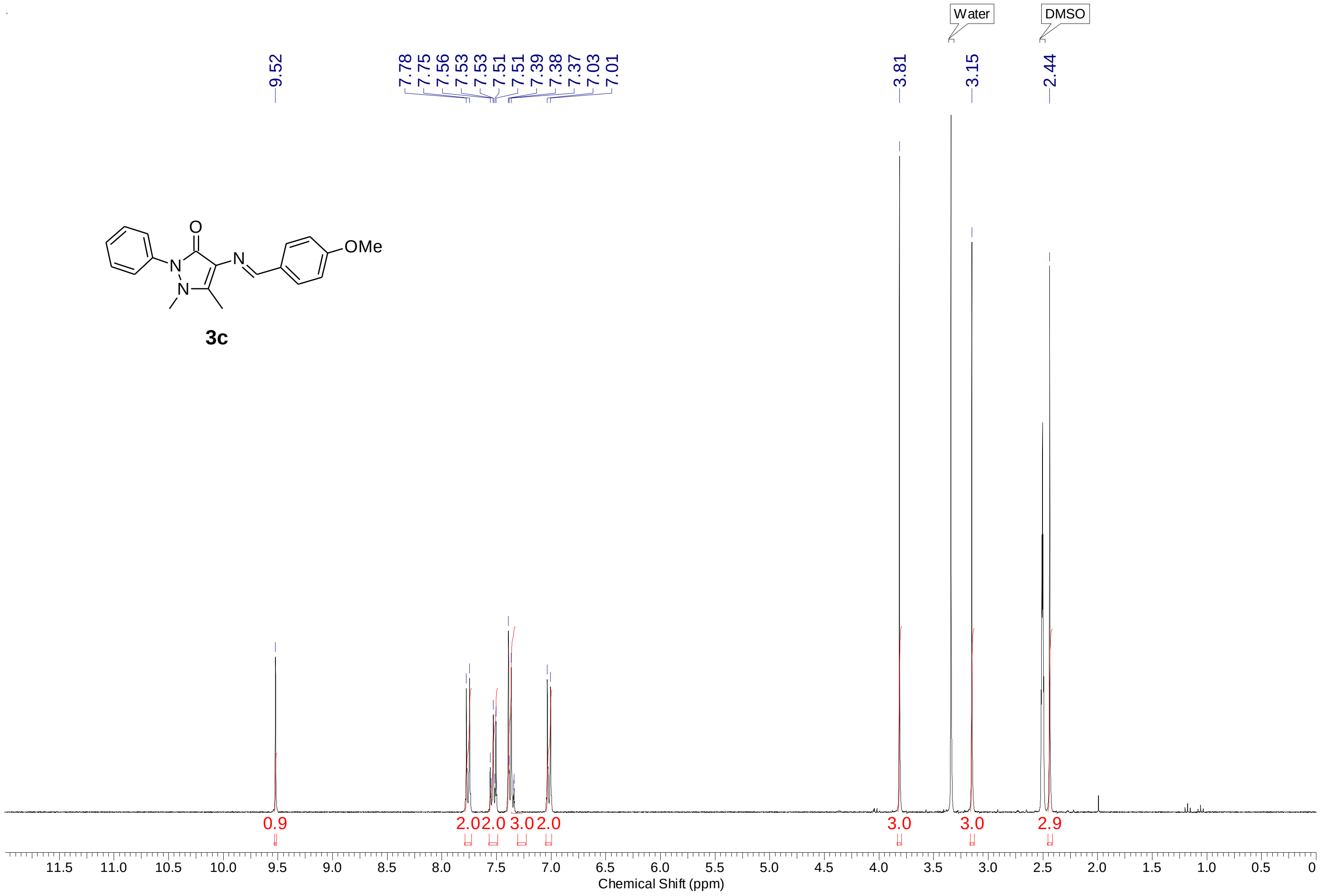

Figure S3. ^{1}H NMR spectrum of compound **3c**.

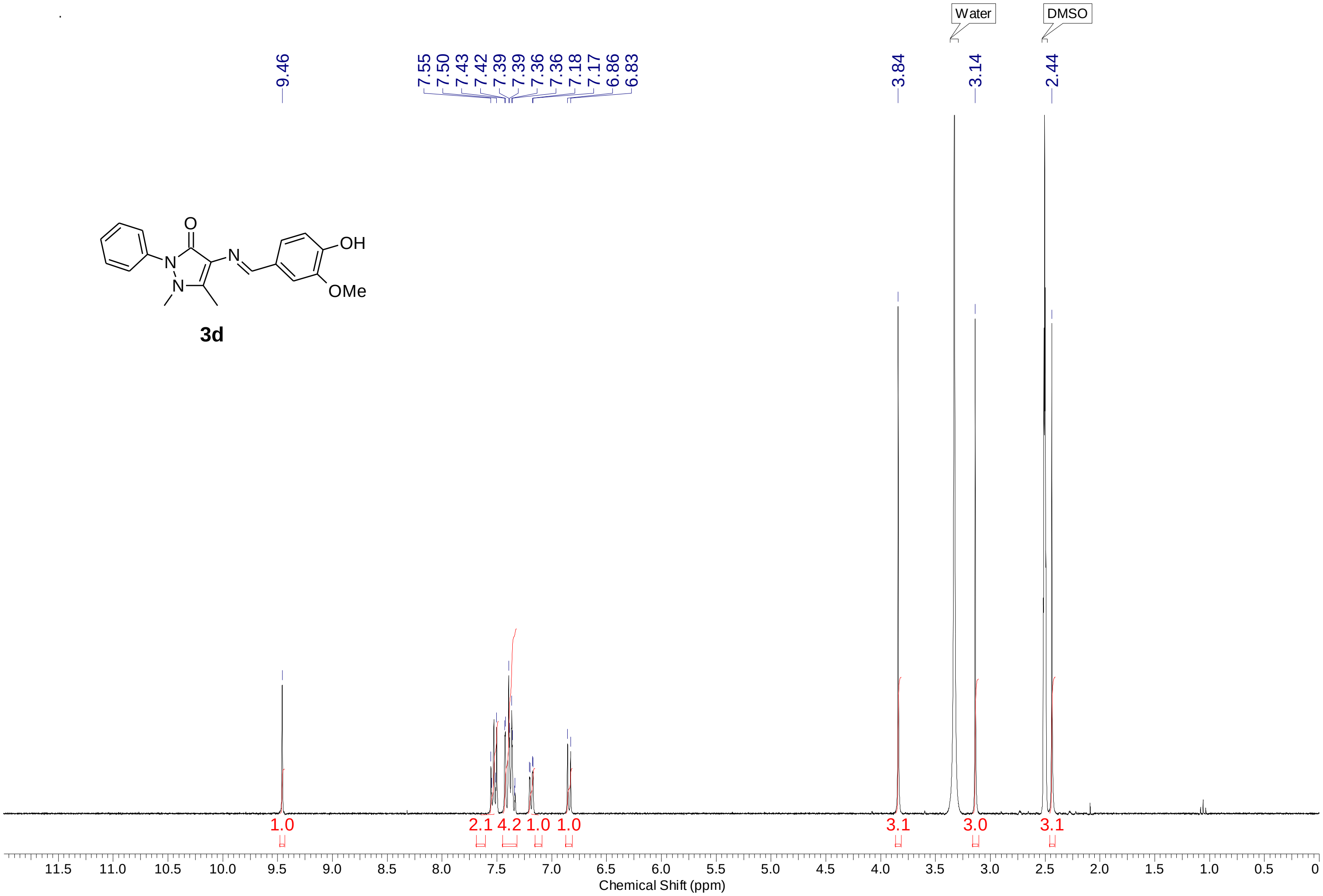

Figure S4. ^{1}H NMR spectrum of compound **3d**.

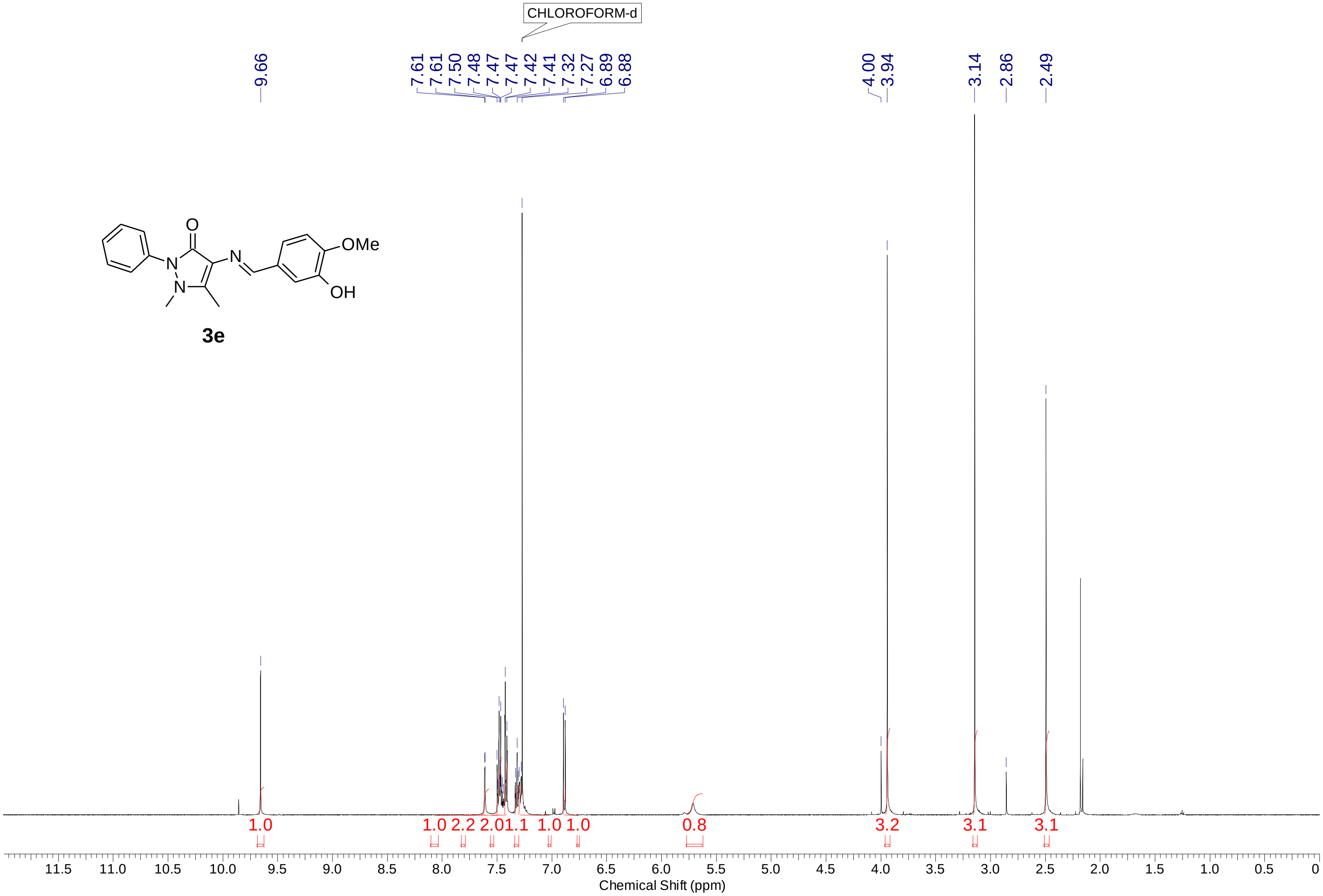

3e

Figure S5. ^{1}H NMR spectrum of compound **3e**.

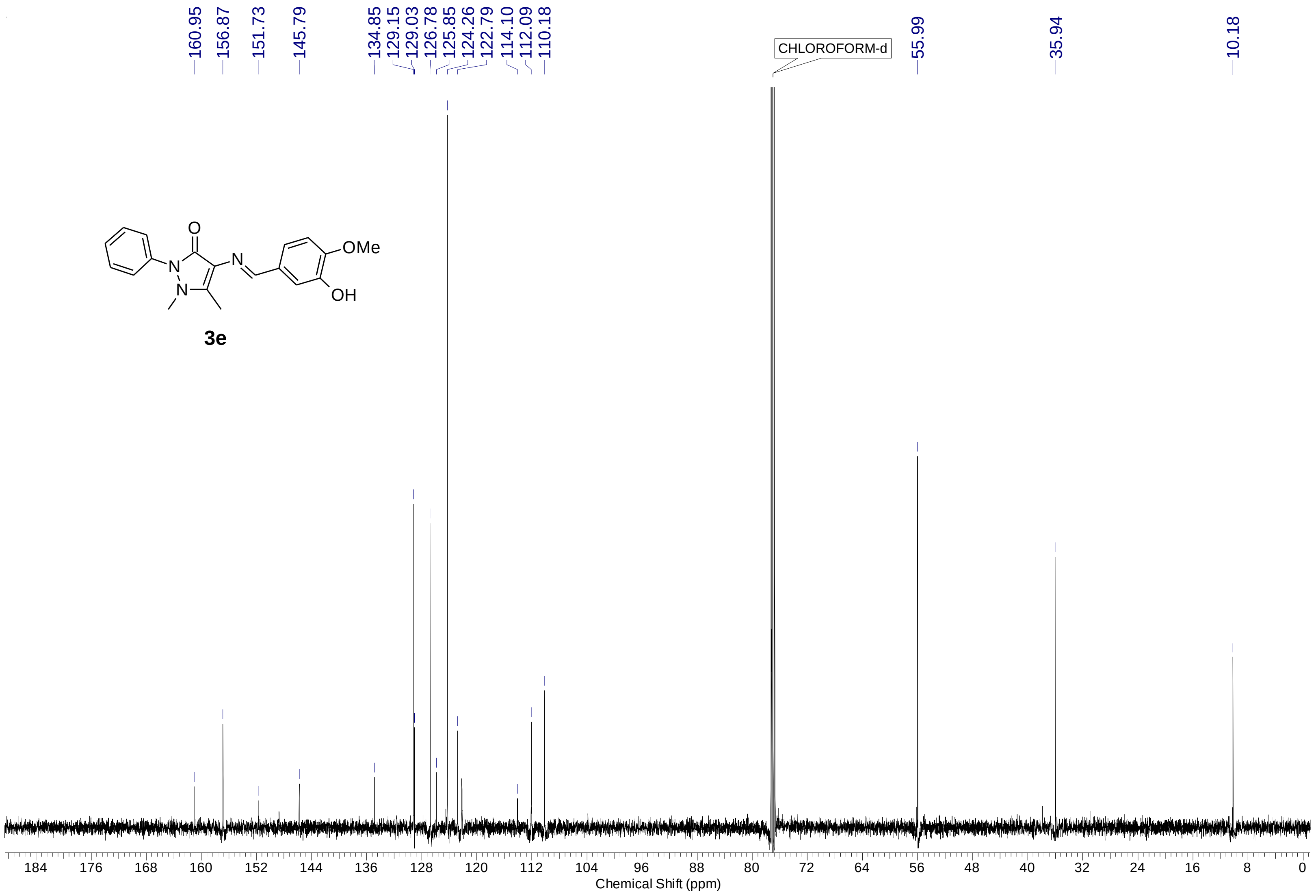

Figure S6. ^{13}C NMR spectrum of compound **3e**.

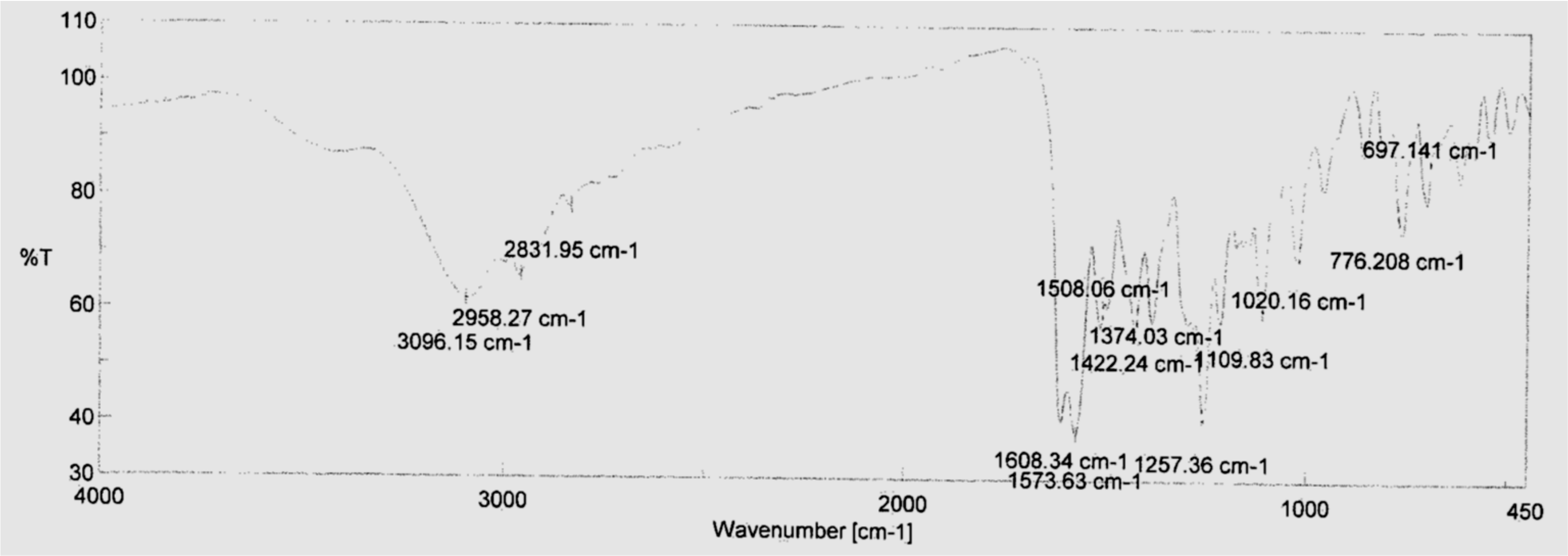

Figure S7. IR spectrum of compound **3e**.

Elemental Composition Report

Single Mass Analysis

Tolerance = 10.0 mDa / DBE: min = -2.0, max = 1000.0
Element prediction: Off
Number of isotope peaks used for i-FIT = 3

Monoisotopic Mass, Even Electron Ions
60 formula(e) evaluated with 3 results within limits (up to 19 closest results for each mass)
Elements Used:
C: 0-100 H: 0-200 N: 3-3 O: 0-30
24-Jun-2016
jhm-24jun16-77 284 (5.252) Cn (Cen,7, 50.00, Ar); Sm (SG, 3x5.00); Sb (12,5.00)

TOF MS ES+
6.54e+003

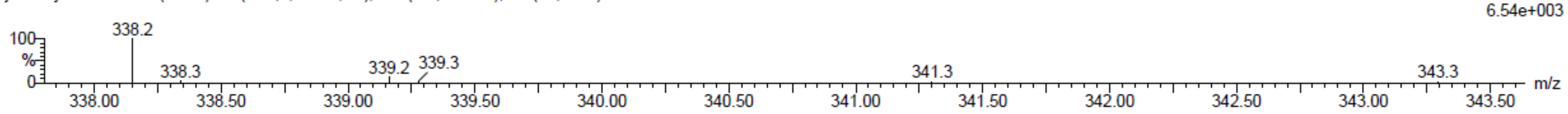

Minimum:				-2.0		
Maximum:		10.0	10.0	1000.0		
Mass	Calc. Mass	mDa	PPM	DBE	i-FIT	Formula
338.1506	338.1505	0.1	0.3	11.5	n/a	C19 H20 N3 O3
	338.1563	-5.7	-16.9	2.5	n/a	C12 H24 N3 O8
	338.1411	9.5	28.1	-1.5	n/a	C8 H24 N3 O11

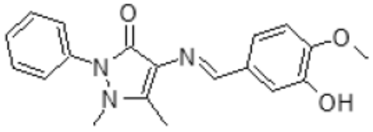

[M+H]+ = 338.149918 Da

Figure S8. HRMS of compound **3e**.

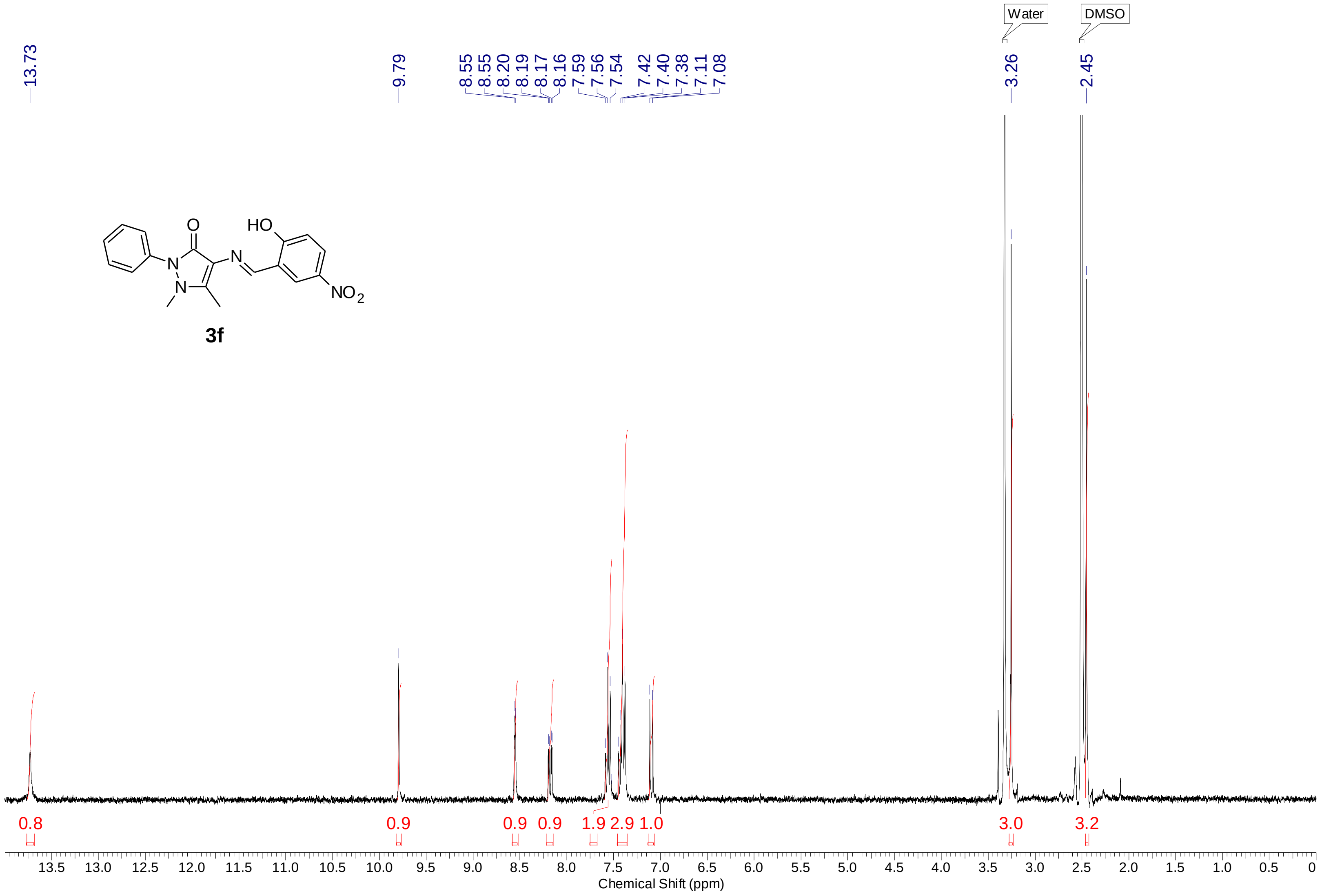

Figure S9. ^{1}H NMR spectrum of compound **3f**.

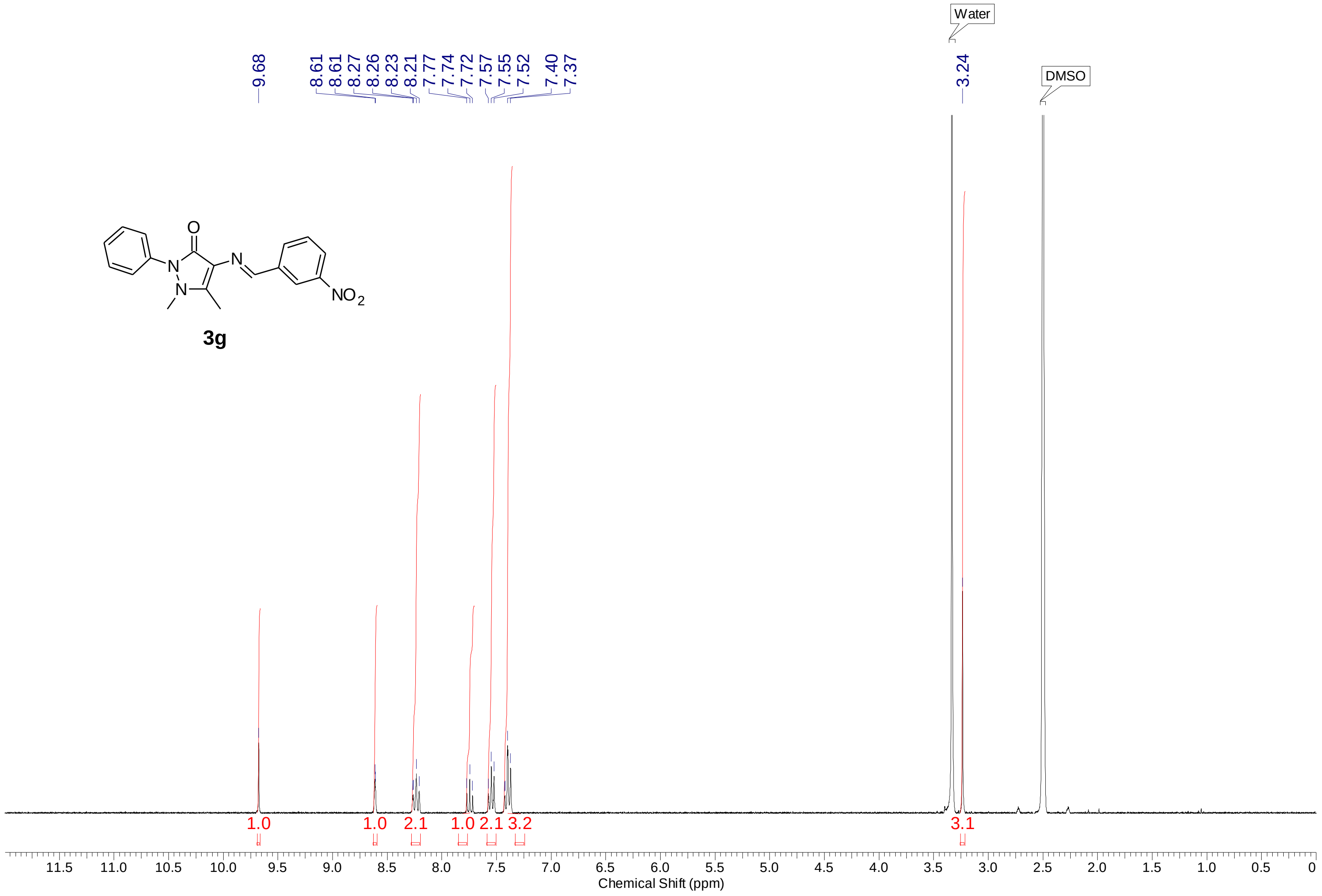

Figure S10. ^{1}H NMR spectrum of compound **3g**.

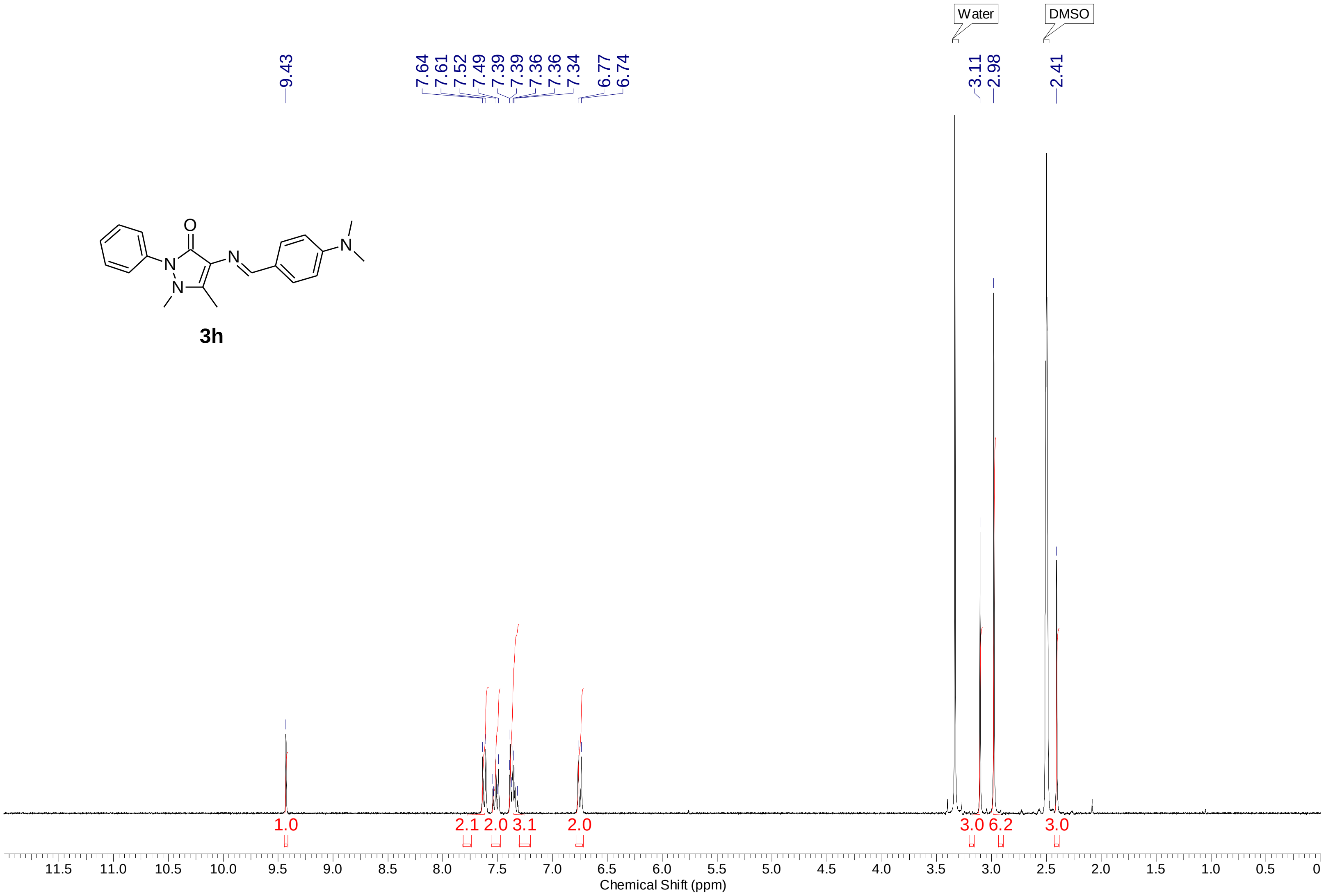

Figure S11. ^{1}H NMR spectrum of compound **3h**.

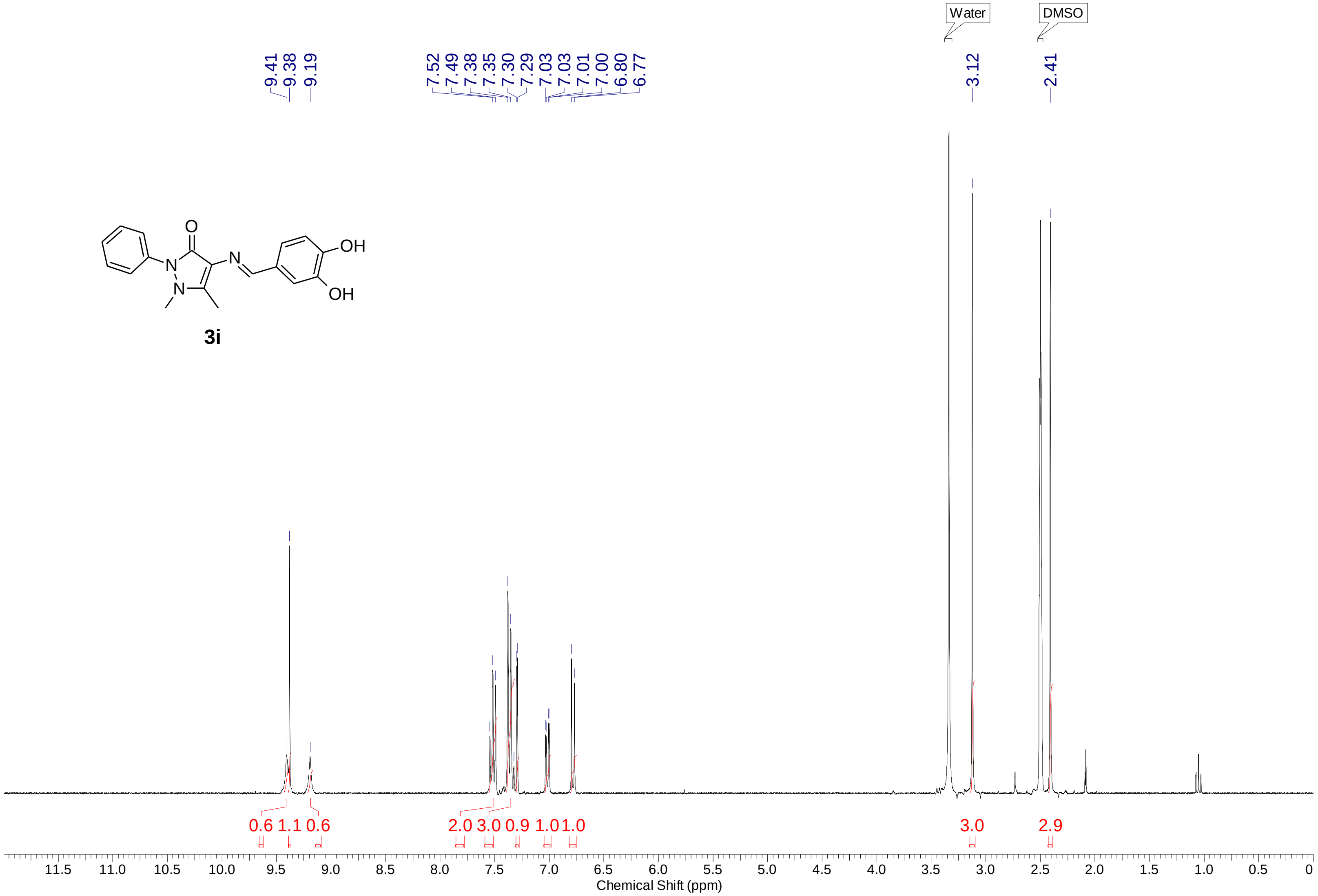

Figure S12. ^{1}H NMR spectrum of compound **3i**.

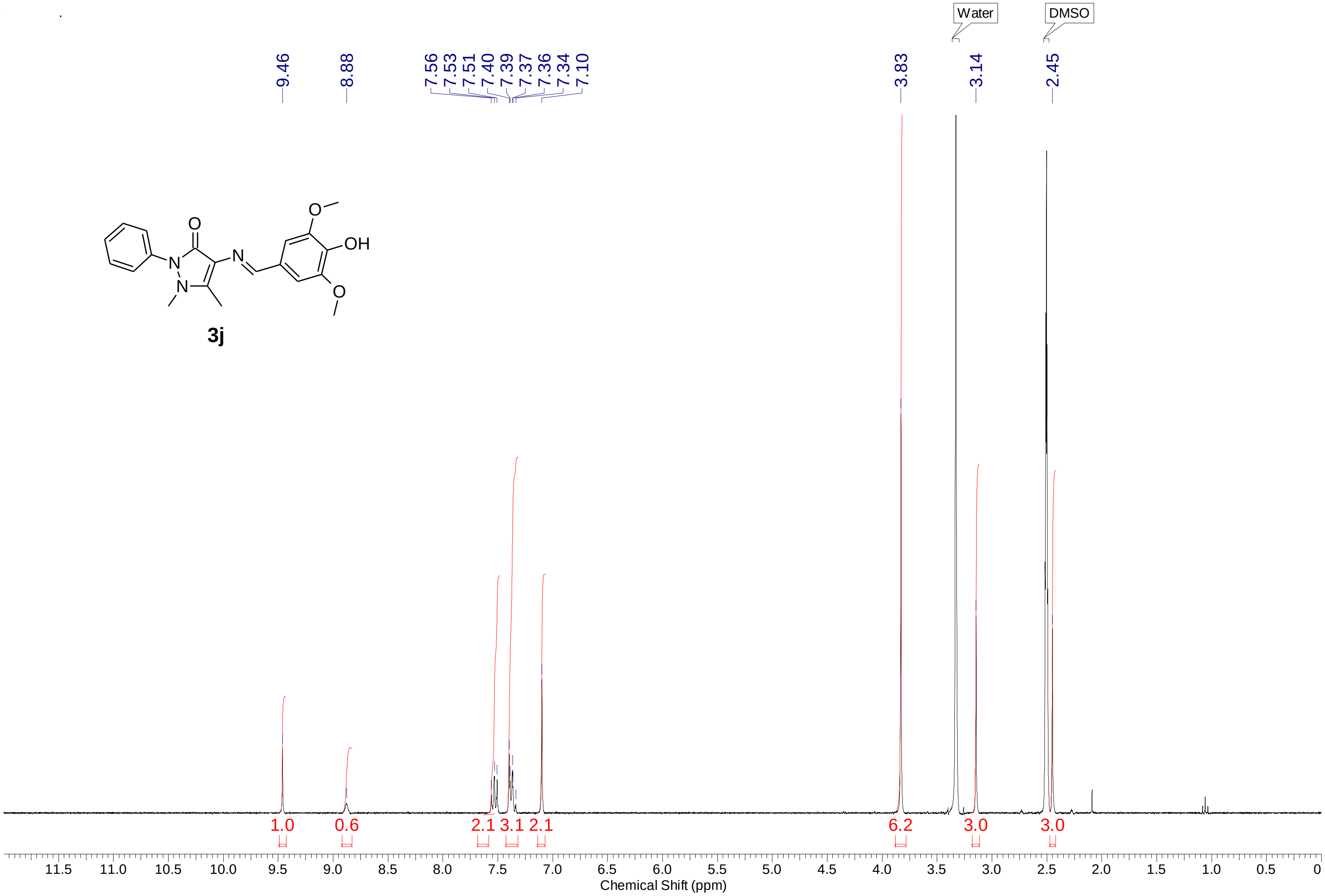

Figure S13. ^{1}H NMR spectrum of compound **3j**.

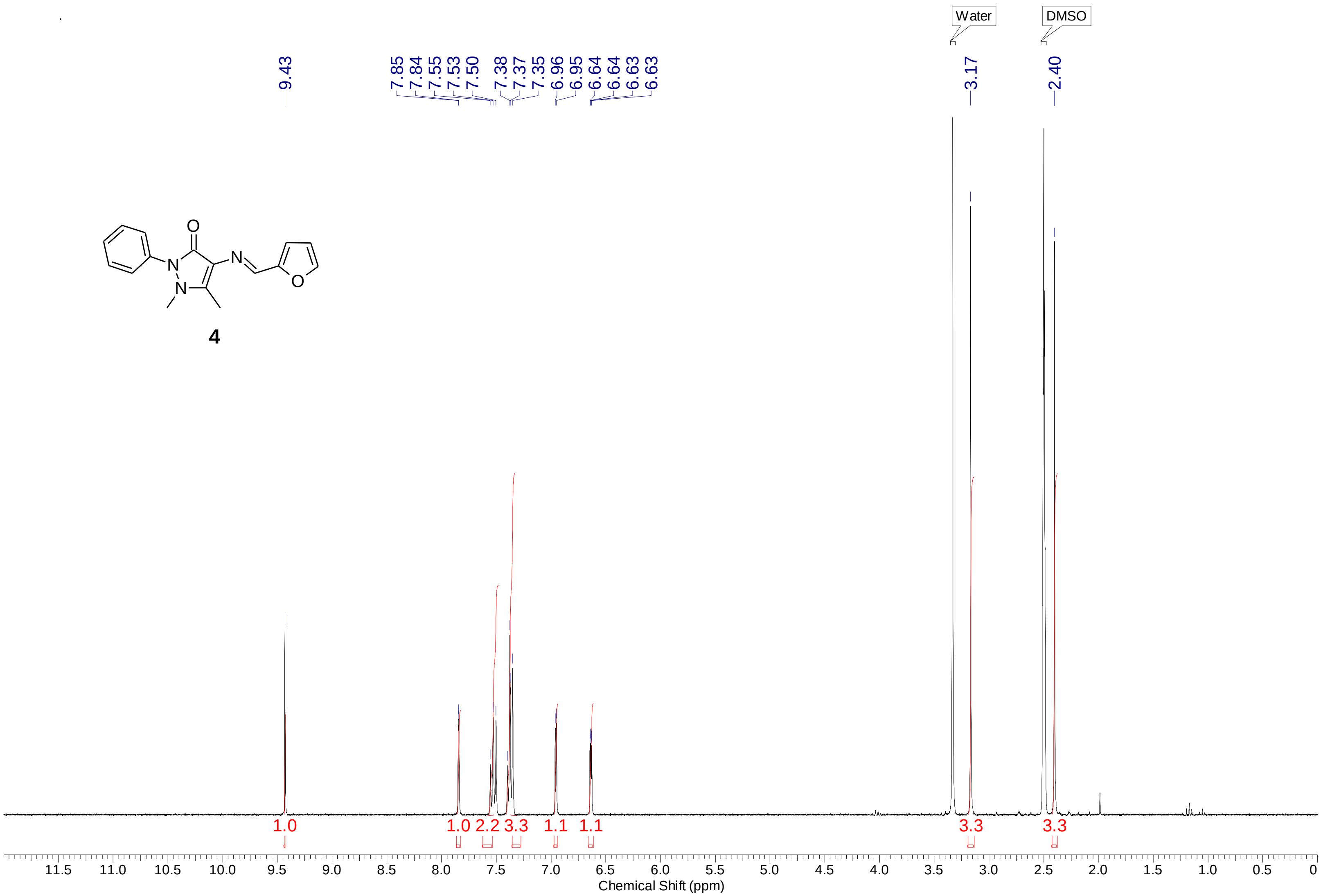

Figure S14. ^{1}H NMR spectrum of compound **4**.

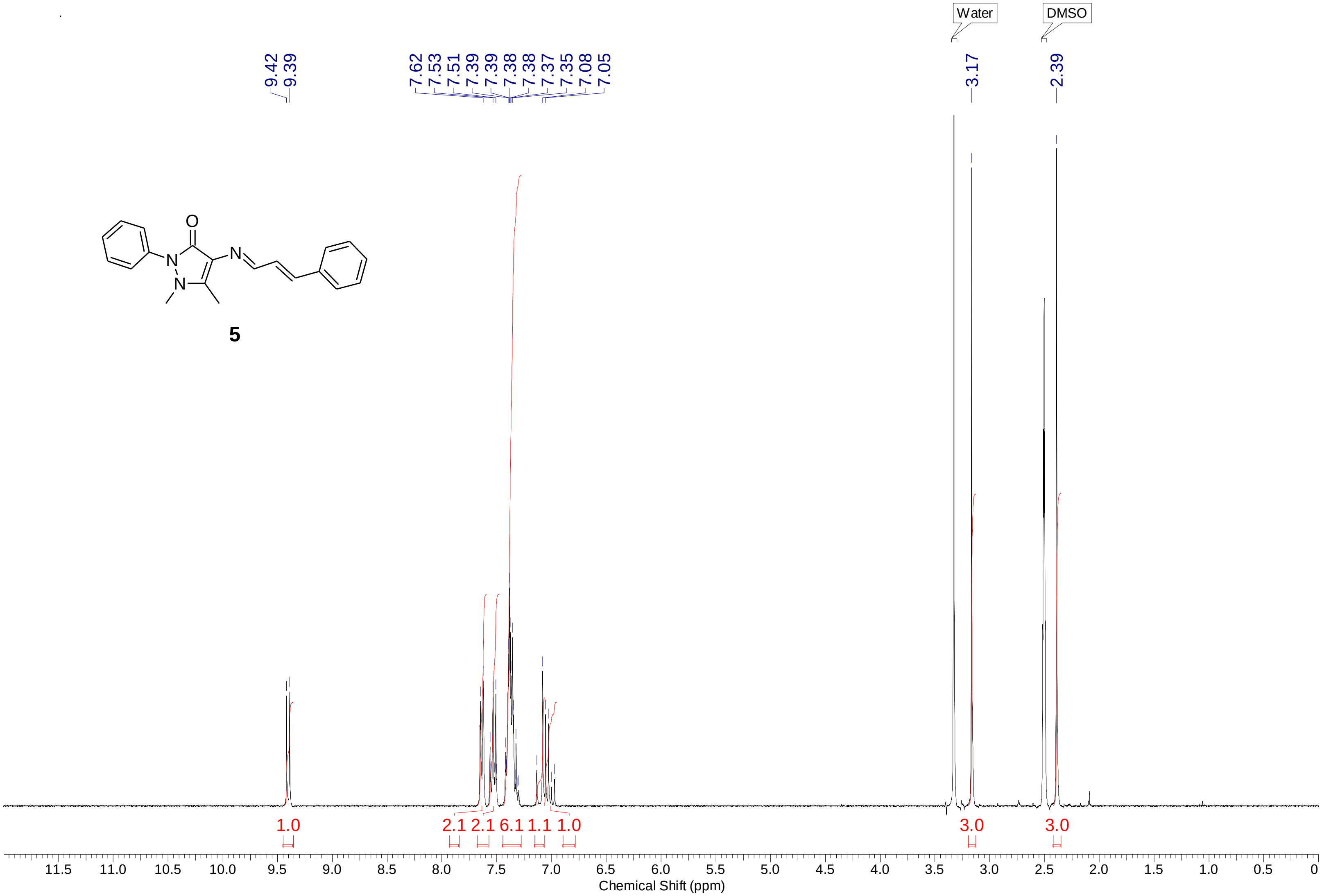

Figure S15. ^{1}H NMR spectrum of compound **5**.

Supplementary figure S16. Evaluation of –cide/-static effect

A. Fungicide effect. *S. cerevisiae* (W303) exposed to Schiff base **5**

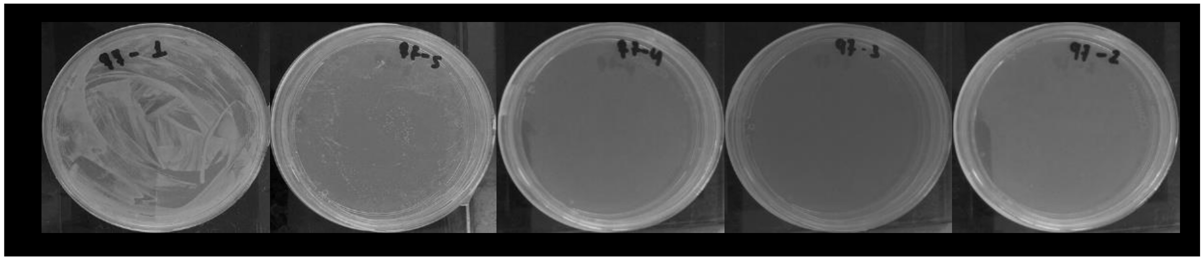

Control (2% DMSO) | 50 μg/mL | 100 μg/mL | 200 μg/mL | 400 μg/mL

B. Bacteriostatic and fungistatic effect. a. *Escherichia coli* exposed to Schiff base **3d**; b. *Enterococcus faecalis* exposed to Schiff base **3a**; c. *Candida albicans* exposed to Schiff base **3b**; d. *Candida albicans* exposed to Schiff base **3f**; e. *Aspergillus niger* exposed to Schiff base **3f**; f. *Staphylococcus aureus* exposed to Schiff base **3f**.

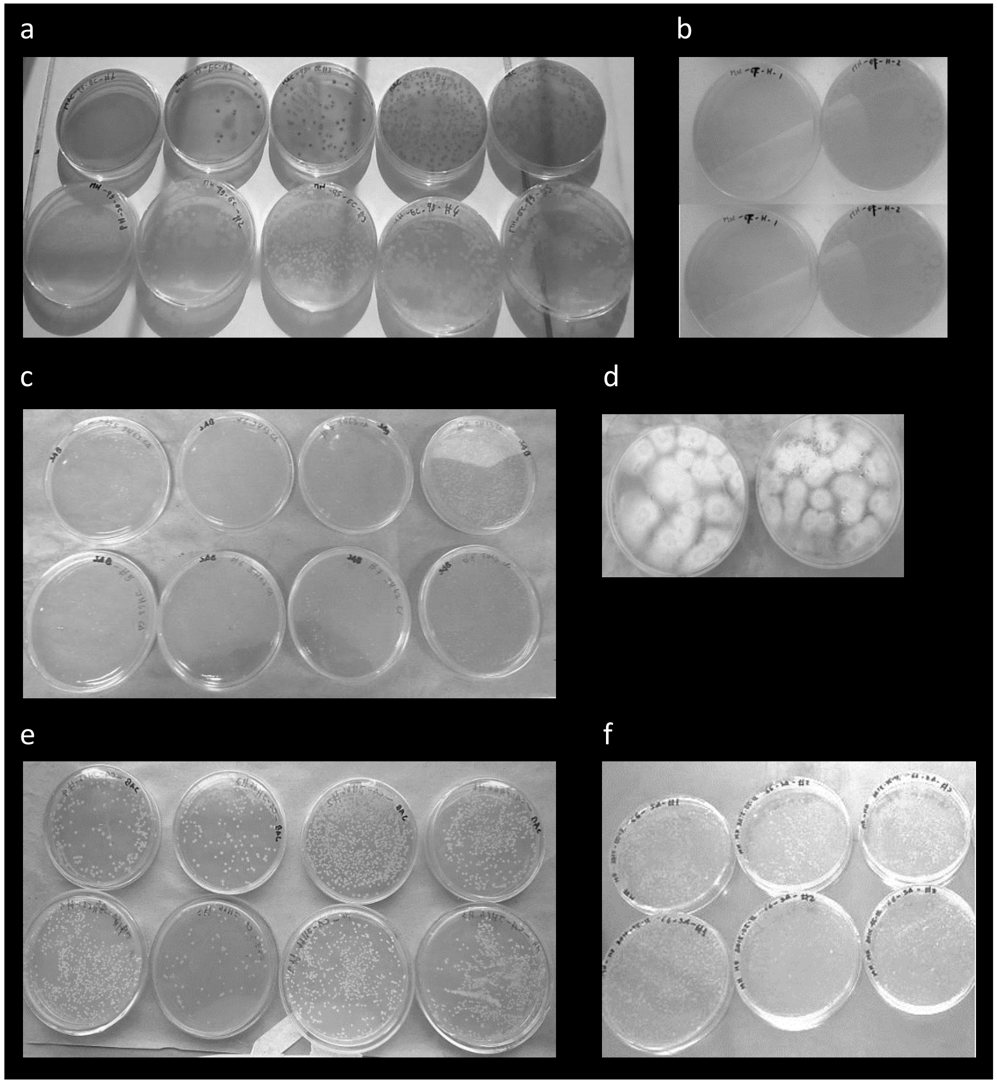

Supplemental Table S1. Theoretical prediction of ADME properties[a] of some drug used in chemotherapy.

Compound	MW	HBA	HBD	nrotb	PSA	M	T	RE	I	cLogP	cLogS	DL	DS	IARC[b]
Cyclophosphamide	261.09	4	1	4	51.38	high	high	high	none	0.73	-1.90	-10.32	0.10	1
Methotrexate	454.45	13	5	9	210.54	none	high	none	none	-1.23	-3.77	-7.59	0.22	3
Fluorouracil	130.08	4	2	0	58.20	high	high	high	high	-0.84	-1.77	-4.50	0.06	3
Doxorubicin	543.52	12	6	5	206.07	none	none	none	high	0.17	-4.51	6.65	0.33	NR
Procarbazine	221.3	4	3	5	53.16	high	high	high	none	0.24	-2.41	3.86	0.20	2A
Prednisolone	360.45	5	3	2	94.83	none	none	none	none	1.14	-2.96	4.10	0.85	NR
Bleomycin	1415.57	38	20	36	683.55	none	none	none	none	-9.96	-2.70	5.30	0.48	2B
Etoposide	588.56	13	3	5	160.83	none	none	none	none	0.67	-3.95	-1.98	0.30	1
Cisplatin	300.05	2	2	0	55.28	none	none	none	none	0.00	-1.93	-3.18	0.49	2A
Epirubicin	543.52	12	6	5	206.07	none	none	none	high	0.17	-4.51	6.65	0.33	NR
Capecitabine	359.35	9	3	6	120.69	high	none	low	low	0.47	-3.37	-21.33	0.16	NR
Folinic acid	473.44	14	7	9	215.55	none	none	none	none	-2.21	-4.08	-6.77	0.34	NR
Temozolomide	194.15	8	1	1	105.94	high	high	high	none	-0.50	-1.90	-1.80	0.12	NR
Anastrozole	293.37	5	0	4	78.29	none	high	low	none	2.61	-4.17	-4.16	0.19	NR

[a] MW: molecular weight; HBA: number of hydrogen bond acceptor; HBD: number of hydrogen bond dador; nrotb: number of rotatable bonds; PSA: polar surface area; M: mutagenicity; T; tumorigenicity; RE: reproductive effect; I: irritant effect; cLogP: logarithm of compound partition coefficient between n-octanol and water; cLogS: logarithm of compound aqueous solubility; DL: drug-likeness; DS: drug score. VR: voriconazole; CP: ciprofloxacin.

[b] Group of classification of IARC; NR = no reported.
